# Topological phases of quantized light

**DOI:** 10.1093/nsr/nwaa196

**Published:** 2020-08-31

**Authors:** Han Cai, Da-Wei Wang

**Affiliations:** Interdisciplinary Center for Quantum Information and State Key Laboratory of Modern Optical Instrumentation, Zhejiang Province Key Laboratory of Quantum Technology and Device and Department of Physics, Zhejiang University, Hangzhou 310027, China; Interdisciplinary Center for Quantum Information and State Key Laboratory of Modern Optical Instrumentation, Zhejiang Province Key Laboratory of Quantum Technology and Device and Department of Physics, Zhejiang University, Hangzhou 310027, China; CAS Center for Excellence in Topological Quantum Computation, University of Chinese Academy of Sciences, Beijing 100190, China

**Keywords:** topological phases, Su-Schriefer-Heeger model, Jaynes-Cummings model, strain-induced magnetic field, Haldane model

## Abstract

Topological photonics is an emerging research area that focuses on the topological states of classical light. Here we reveal the topological phases that are intrinsic to the quantum nature of light, i.e. solely related to the quantized Fock states and the inhomogeneous coupling strengths between them. The Hamiltonian of two cavities coupled with a two-level atom is an intrinsic one-dimensional Su-Schriefer-Heeger model of Fock states. By adding another cavity, the Fock-state lattice is extended to two dimensions with a honeycomb structure, where the strain due to the inhomogeneous coupling strengths of the annihilation operator induces a Lifshitz topological phase transition between a semimetal and three band insulators within the lattice. In the semimetallic phase, the strain is equivalent to a pseudomagnetic field, which results in the quantization of the Landau levels and the valley Hall effect. We further construct an inhomogeneous Fock-state Haldane model where the topological phases can be characterized by the topological markers. With *d* cavities being coupled to the atom, the lattice is extended to *d* − 1 dimensions without an upper limit. In this study we demonstrate a fundamental distinction between the topological phases in quantum and classical optics and provide a novel platform for studying topological physics in dimensions higher than three.

## INTRODUCTION

Topological phases of matter have been extensively investigated not only for electrons [1–5], but also for neutral atoms [6,7], photons [8,9] and phonons [10,11]. However, regarding whether the topological phases are quantum or classical, there is a fundamental difference between electrons and photons (and similarly phonons). While the topological phases of electrons are intrinsically quantum, i.e. based on the Schrödinger equation and fermionic statistics of electrons, the topological phases of light originating from the analogy between the Maxwell and Schrödinger equations can be explained in the framework of classical optics [8,9,12]. Although in lattices of resonators [13] a quantized field formulation of light is used to facilitate the calculation of the chiral edge modes in parallel with those of electrons, the topological phases have no quantum signature and can be demonstrated with classical light. A natural question is whether the second quantization of light embeds new topological phases that are fundamentally distinct from those classical ones. Such topological phases of quantized light can bring together two relatively unrelated areas, quantum electrodynamics and topological matter, and provide a new perspective on the relations between different topological phases in condensed matter physics.

Early discoveries that require field quantization include black-body radiation, the Lamb shift [14] and the Casimir effect [15]. Black-body radiation reveals the quantized eigenstates of light, i.e. the Fock states denoted by |*m*〉 with *m* being the number of photons in the states. The latter two result from quantum fluctuations of the vacuum state |0〉. The quantized Fock states have profound consequences in atom-photon interactions, such as the collapse and revival of Rabi oscillations [16–18] when a two-level atom is resonantly coupled to a coherent field, i.e., in the Jaynes-Cummings (JC) model [19]. This phenomenon is due to the quantum interference between the Rabi oscillations of the atom coupled to different Fock states |*m*〉, which have discrete Rabi frequencies proportional to $\sqrt{m}$. This is reminiscent of the Landau levels of electrons near the Dirac cones of graphene in a magnetic field [20,21], which also follows the same scaling. In this paper, among other interesting connections between the JC model and the topological phases in condensed matter physics, we reveal the surprising relation between the $\sqrt{m}$ scaling of the Rabi frequencies and the Landau levels through a lattice composed by Fock states, coined the Fock-state lattice (FSL) [22].

Before we sketch the basic structure of the FSL, we emphasize that the quantization of the light field allows arbitrarily large lattices to be synthesized by only a few light modes. The Fock states of *d* modes of photons are |*n*_1_, *n*_2_, …, *n*_*d*_〉, where *n*_*j*_ = 0, 1, 2, … is the photon number in the *j*th mode. Each mode offers an independent degree of freedom. Our strategy is to use this many-body Fock space of a few bosonic modes to simulate the single-particle Hilbert space of either bosons or fermions. We introduce the FSL with the Hamiltonian of a multimode JC model (ℏ = 1),

(1) \begin{eqnarray*} H&=&\sum _{j=1}^{d}\nu _j a_j^\dagger a_j+\frac{\omega \sigma _z}{2}\nonumber\\ &&+\,\,\frac{g}{\sqrt{d}\, }\sum _{j=1}^{d} \left(a_j^\dagger +a_j\right)(\sigma ^-+\sigma ^+), \end{eqnarray*}

where σ^−^ = |↓〉〈↑| and σ^+^ = |↑〉〈↓| are the lowering and raising operators of the two atomic states |↑〉 and |↓〉 with transition frequency ω, *a*_*j*_ and $a_j^\dagger$ are the annihilation and creation operators of the *j*th mode with frequency ν_*j*_ and $g/\sqrt{d}$ is the coupling strength between the photons and the atom. Assuming that ν_*j*_ = ω, we make the rotating-wave approximation and obtain the following Hamiltonian in the interaction picture:

(2) \begin{equation*} {H=\frac{g}{\sqrt{d}\, }\sum _{j=1}^{d} \left(a_j^\dagger \sigma ^-+\sigma ^+a_j\right)}. \end{equation*}

**Figure 1.**
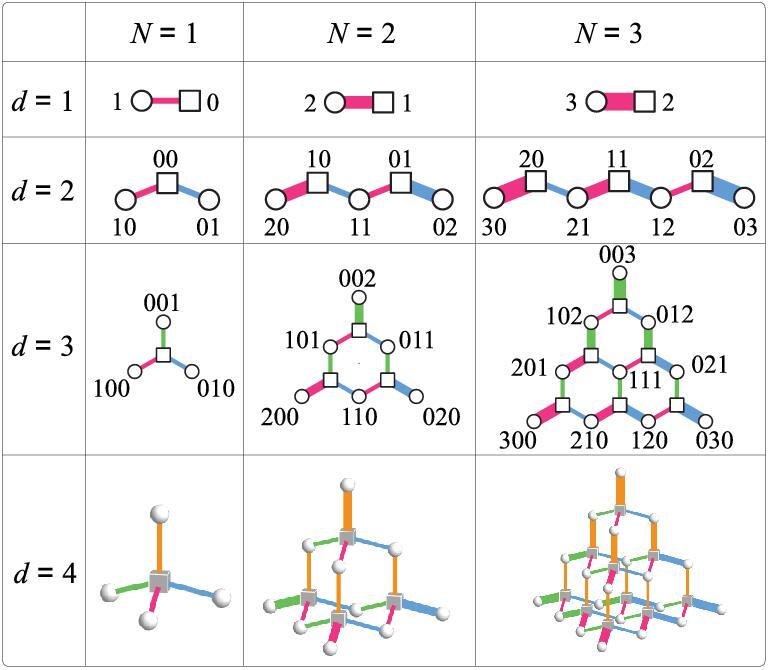
Fock-state lattices in *d* − 1 dimensions of the Hamiltonian in Equation (2) with total excitation number *N*. The squares/circles denote the states |↑/↓, *n*_1_, *n*_2_, …, *n*_*d*_〉 in the sublattices characterized by the |↑〉/|↓〉 atomic states. The numbers labeling the lattice sites are the photon numbers *n*_1_*n*_2_…*n*_*d*_ in the corresponding states. For clarity, we only label the photon numbers in the |↓〉 sublattice for *d* = 3 and hide all photon numbers for *d* = 4. The widths of the lines connecting neighboring sites are proportional to the magnitudes of the coupling strengths between them.

This Hamiltonian conserves the total number of excitation $N=\sum _j a_j^\dagger a_j +(\sigma _z+1)/2$, where σ_$z$_ = |↑〉〈↑| − |↓〉〈↓| is the $z$ component of the Pauli matrices of the atom. We have two ways to look into the Hamiltonian in Equation (2). Each state |↑, *n*_1_, *n*_2_, …, *n*_*d*_〉 is coupled to *d* neighbors |↓, *n*_1_, *n*_2_, …, *n*_*j*_ + 1, …, *n*_*d*_〉 (where *j* = 1, 2, …, *d*) with coupling strengths proportional to $\sqrt{n_j+1}$, forming a bipartite (corresponding to the two states of the atom) FSL with site-dependent coupling strengths in synthetic *d* − 1 dimensions [23] (see Fig. 1). From another perspective, by combining the *a* modes to form a collective mode $b=\sum _j a_j/\sqrt{d}$, the Hamiltonian becomes the single-mode JC model, which is analytically solvable. Combination of these two pictures enables us to study the topological phases of the FSL.

Before laying out the details, we first highlight a couple of distinctive features of the FSL. They are lattices of quantum states instead of modes and have natural edges based on the fact that the photon numbers in Fock states have a lower limit zero, i.e. the existence of the vacuum state. An advantage of the FSL is that their dimensions have no upper limit, providing a unique platform to investigate topological phases in dimensions higher than three. However, we must take special care of the coupling strengths, which vary locally depending on the photon numbers in the Fock states. Here we show that, for the one-dimensional (1D) FSL with *d* = 2, the variation of the coupling strengths results in the topological zero-energy state between two different topological phases of the Su-Schriefer-Heeger (SSH) model [24,25]. In two dimensions with *d* = 3, the variation of the coupling strengths is equivalent to a strain field in the honeycomb lattice, which leads to a Lifshitz topological phase transition between a semimetal and three band insulators within the FSL [21], as well as a strain-induced pseudomagnetic field [26,27] in the semimetallic phase. The pseudomagnetic field results in quantized Landau levels and provides the basis to observe the valley Hall effect [28–30] and construct a Fock-state Haldane model [2], where the topological phases are characterized by topological markers [31,32]. The FSL can be extended to higher dimensions to study the topological phases unachievable in real space [33–37]. It also provides a solution to design finite lattices with exactly quantized energy levels [38,39].

## RESULTS

### 1D Fock-state SSH model

We first show the relation between the SSH model and the 1D FSL with the Hamiltonian

**Figure 2.**
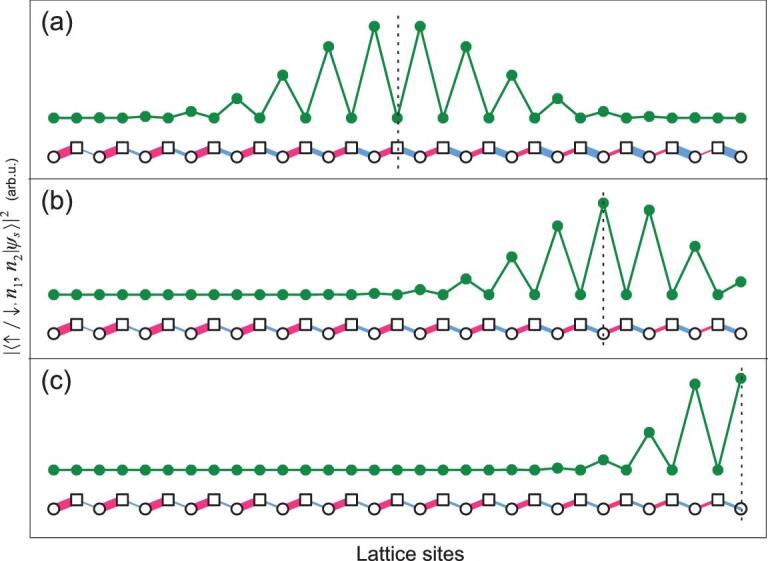
The probability distribution of the topological zero-energy state |ψ_*s*_〉 in the 1D Fock-state SSH model. The lattices are plotted in the same way as in Fig. 1 with *N* = 15. The probability distribution of |ψ_*s*_〉 is plotted above the corresponding lattice sites. The ratio *u*_1_/*u*_2_ = 1 (a), 2 (b) and 4 (c). The neighboring probabilities are connected by straight lines as a visual guide. The vertical dashed lines denote the boundary between two topological phases of the SSH model.

(3) \begin{equation*} H_1=g\sigma ^+(u_1 a_1+u_2 a_2) +{\rm H.c.}, \end{equation*}

where *u*_1_ and *u*_2_ are real positive numbers satisfying $u_1^2+u_2^2=1$. In Fig. 2(a)–(c), we illustrate the FSL with *N* = 15 in the basis of |↓/↑, *n*_1_, *n*_2_〉 for different values of *u*_1_/*u*_2_. The connection between this lattice and the topological SSH model is endorsed by the variation of the coupling strengths due to the property of the annihilation operator, $a|n\rangle =\sqrt{n}|n-1\rangle$. For $u_1=u_2=1/\sqrt{2}$, the lattice is equally divided into two parts. On the left side, the coupling strengths of *a*_1_ are larger than those of *a*_2_, contrary to their relation on the right side. Accordingly, these two parts are in two different topological phases of the SSH model, which is evident from the topological zero-energy state at the boundary, as shown in Fig. 2(a)–(c). We can tune *u*_1_ and *u*_2_ to move the zero-energy state, which is always located at the boundary satisfying $u_1\sqrt{n_1}=u_2\sqrt{n_2}$ (see the online supplementary material). When $u_1>\sqrt{N}u_2$ (or $u_2>\sqrt{N}u_1$), there is only one topological phase and the zero-energy state is on one of the ends of the lattice.

The eigenenergies and eigenstates of Equation (3) are analytically obtained by recombining *a*_1_ and *a*_2_ to form a bright mode *b*_1_ = *u*_1_*a*_1_ + *u*_2_*a*_2_ and a dark mode *b*_2_ = *u*_2_*a*_1_ − *u*_1_*a*_2_. Only the bright mode is coupled with the atom. The corresponding eigenstates are $|\psi _{m}^\pm \rangle =(|\!\downarrow ,m,N-m\rangle _b \,\, \pm\, |\!\uparrow,$$m-1,N-m\rangle _b)/\sqrt{2}$, where *m* = 1, 2, …, *N* in | … 〉_*b*_ is the photon number in the *b*_1_ mode. The eigenstate with *m* = 0 is the topological zero-energy state |ψ_*s*_〉 = |↓, 0, *N*〉_*b*_, which has zero energy and only occupies the |↓〉 sublattice. It is interesting to note that this bimodal JC model has also been related to the topological properties of the Jahn-Teller system [40].

### Effective strain, pseudomagnetic field and Landau levels in the 2D FSL

The lattice is extended to two dimensions by adding a third cavity mode in the Hamiltonian

(4) \begin{equation*} H_2=\frac{g}{\sqrt{3}\, }\sigma ^+(a_1+a_2+a_3)+{\rm H.c.} \end{equation*}

**Figure 3.**
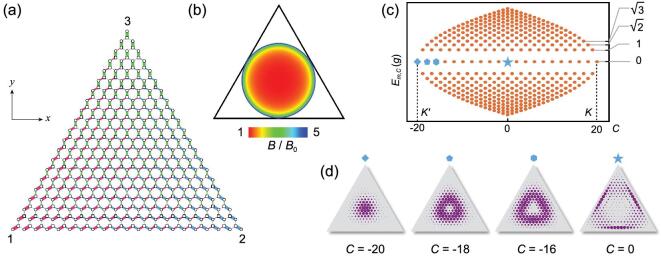
Two-dimensional Fock-state lattice with an effective pseudomagnetic field and Landau levels. (a) The Fock-state lattice of the Hamiltonian *H*_2_ in Equation (4) for *N* = 20. The three numbers 1, 2, 3 denote the states at the vertices with all *N*  photons in modes *a*_1_, *a*_2_ and *a*_3_. The coupling strengths *t*_1_, *t*_2_ and *t*_3_ are drawn with red, blue and green lines with widths proportional to the strengths. (b) The distribution of the effective pseudomagnetic field due to the variation of the coupling strengths within the incircle, evaluated from Equation (15). Outside of the incircle the strain induces a band gap. (c) The band structure of the generalized Landau levels with eigenenergies $E^\pm _{m,C}=\pm \sqrt{m}g$ for the eigenstates $|\psi ^\pm _{m,C}\rangle$. (d) The wavefunctions of the eigenstates in the zeroth Landau level |ψ_0, *C*_〉 for *C* = −20, −18, −16 and 0, labeled with a diamond, pentagon, hexagon and star in (c).

The Fock states |↑/↓, *n*_1_, *n*_2_, *n*_3_〉 form a honeycomb lattice with triangular boundaries on which one of the cavity modes is in the vacuum state, as shown in Fig. 3(a). All photons are in one cavity at the three vertices, which are labeled with the corresponding cavity numbers. The inhomogeneous coupling strengths introduce an effective strain in the lattice. We first note that in the center of the lattice the strain is relatively small, while approaching the vertices the strain becomes drastic. When the strain is small such that [27]

(5) \begin{equation*} |t_1-t_2|< t_3 < |t_1+t_2| \end{equation*}

with $t_j=g\sqrt{n_j}/\sqrt{3}$ being the coupling strength of mode *a*_*j*_, the strain field is equivalent to a pseudomagnetic field leading to quantized Landau levels [26,27,38], which have been experimentally implemented in graphene [41]. The lattice sites that satisfy Equation (5) are in the incircle of the FSL, i.e. where (see Fig. 3(b) and the Methods section)

(6) \begin{equation*} n_1^2+n_2^2+n_3^2<\frac{N^2}{2}. \end{equation*}

Beyond the incircle the strain is so large that a band gap opens and we cannot regard the strain as a simple pseudomagnetic field. A Lifshitz topological phase transition between a strained semimetal and a band insulator [21] occurs on the incircle of the 2D FSL.

We first evaluate the strength of the pseudomagnetic field near the center of the FSL. This can be done by comparing the eigenenergies of Equation (4) and those of the Landau levels in real graphene. The Landau levels are characterized by $\pm \sqrt{mB}$ scaling near the Dirac cone, with *B* being the strength of the magnetic field, *m* being the index of the Landau levels and ± for the conduction and valence bands [21]. The eigenenergies of the Hamiltonian *H*_2_ are obtained by recombining the cavity modes to form a collective bright mode, $b_0=(a_1+a_2+a_3)/\sqrt{3}$. The JC model of the *b*_0_ mode coupling with the atom has eigenenergies $\pm \sqrt{m} g$ with $m=\langle b_0^\dagger b_0\rangle$, i.e. in accord with the scaling of the Landau levels in graphene, with effective cyclotron frequency *g*. By recalling the explicit energies of Landau levels in graphene [21] and comparing them with the eigenenergies of *H*_2_, we obtain

(7) \begin{equation*} \pm \sqrt{m}g=\pm \sqrt{2m} \frac{3t_h q}{2l_B}, \end{equation*}

where *t*_*h*_ is the hopping coefficient and *q* is the lattice constant, and the magnetic length $l_B=\sqrt{\hbar /eB}$ with *e* being the electric charge.

At the center of the honeycomb FSL where $\langle a_j^\dagger a_j\rangle \approx N/3$ for *j* = 1, 2, 3, the coupling strengths are $t_1=t_2=t_3=t_h\equiv \sqrt{N}g/3$, which can be regarded as the unstrained background hopping coefficient. The pseudomagnetic field is built upon the deviation of the coupling strengths from *t*_*h*_ due to the variation of the photon numbers. Substituting *t*_*h*_ into Equation (7), we obtain

(8) \begin{equation*} \frac{l_B}{q}=\sqrt{\frac{N}{2}}, \end{equation*}

which is the only relevant quantity to measure the strength of the pseudomagnetic field since both *q* and *l*_*B*_ are fictitious in the FSL. The strength of the corresponding pseudomagnetic field is

(9) \begin{equation*} B_0=\frac{2\hbar }{Neq^2}. \end{equation*}

The fictitious electric charge *e* in *B*_0_ is only an analogous quantity for the convenience of comparison with electrons. All observables in the lattice are independent of *e*. However, to have a general idea of the strength of *B*_0_, we take the lattice constant *q* = 0.14 nm of graphene and obtain *B*_0_ = 6.5 × 10^4^/*N* tesla. For *N* = 20, *B*_0_ is 10 times larger than those demonstrated in graphene [41].

The pseudomagnetic field can only be regarded as approximately uniform near the center of the lattice. The explicit distribution of the pseudomagnetic field is obtained through the valley Hall response (see Equation (15)), or directly from the strain-induced motion of the Dirac cones (see the Methods section). Interestingly, despite the complications of the nonuniform pseudomagnetic field and the topological phase transition on the incircle, all the eigenstates in the 2D FSL are grouped in quantized energy levels with the $\pm \sqrt{m}$ scaling. In the following, we regard these levels as generalized Landau levels of the FSL.

The degeneracy of the eigenstates in the *m*th Landau level is *N* − *m* + 1. To distinguish these states, we introduce the bosonic chirality operator

(10) \begin{equation*} C=b_+^\dagger b_+-b_-^\dagger b_-, \end{equation*}

where $b_\pm =\sum _{j=1}^3 a_j\exp {(\mp i 2j\pi /3)}/\sqrt{3}$ are the annihilation operators of the two dark modes. Here *C* is a good quantum number that plays the role of the lattice momentum in an infinite lattice. It also characterizes the angular momentum carried by the photons in the eigenstates of the FSL. This quantity is an extension of the spin chirality [42] (see the online supplementary material). In graphene, the *K* and *K*′ points correspond to the two maximum momenta in the Brillouin zone [20]. In the finite FSL the points with *C* = *N* and *C* = −*N* are the counterparts of the *K* and *K*′ points. The band structure of the 2D FSL is shown in Fig. 3(c).

The eigenstates in the *m*th Landau level are $|\psi ^\pm _{m,C}\rangle =(| \downarrow , m, m_+, m_-\rangle _b\,\, {\pm}\,\, | \uparrow , m-1, m_+,\hspace*{-4pt}\break m_-\rangle _b)/\sqrt{2}$, where *m*_+_ and *m*_−_ are the photon numbers in the two dark modes. The *N* + 1 eigenstates in the zeroth Landau level are solely composed of |↓〉-sublattice states, |ψ_0, *C*_〉 = |↓, 0, *m*_+_, *m*_−_〉_*b*_, which are the counterparts of the topological zero-energy state in the 1D FSL. We recall that in graphene the electrons in the zeroth Landau level of a real magnetic field occupy only one sublattice at point *K* and the other sublattice at point *K*′ [21]. When the direction of the magnetic field is reversed, the zeroth-Landau-level occupations of the two sublattices at the *K* and *K*′ points are exchanged. Since the strain-induced pseudomagnetic field has opposite signs at the *K* and *K*′ points, the states in the zeroth Landau level of the FSL occupy only the |↓〉 sublattice at both the *K* and *K*′ points [43,44]. Because of the opposite signs of the pseudomagnetic field at the *K*′ and *K* points, *C* can only increase at the *K*′ point and decrease at the *K* point, such that the angular momenta of the eigenstates at these two points can only take positive or negative values when they are counted with respect to their extrema, which is analogous to electrons in magnetic fields with opposite signs [45].

The wavefunctions of the eigenstates can be analytically obtained through an expansion in the Fock states of *a* modes. In Fig. 3(d), we draw several eigenstates in the zeroth Landau level. Near the *K*′ point for *C* = −20, −18 and −16, the eigenwavefunctions resemble those in the zeroth Landau level of a real magnetic field with the symmetric gauge, but with a smaller localization length (see the distribution and phase of the wavefunctions in the online supplementary material). From this point we can also understand the angular momenta *C* of the eigenstates, since they are well defined in the symmetric gauge [45]. When |*C*| decreases, the eigenstate approaches the incircle of the triangular boundary, as shown by |ψ_0, 0_〉 in Fig. 3(d) (see more wavefunctions in the online supplementary material).

### The valley Hall effect

To demonstrate the transport due to the pseudomagnetic field, we can introduce an effective electric field in the lattice and calculate the Hall response of states at points *K* and *K*′. A static electric field induces a linear potential energy of electrons in real space. In the FSL, such a linear potential energy can be introduced by the frequency difference between the cavity modes, e.g.

**Figure 4.**
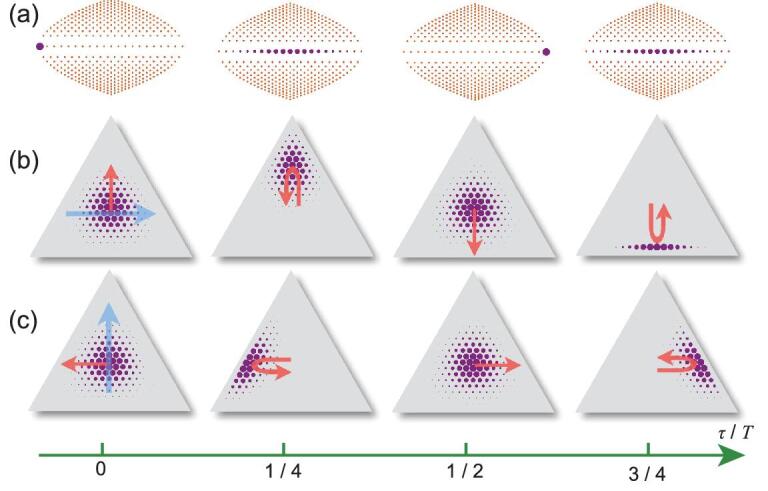
The Bloch oscillation and the valley Hall effect in the zeroth Landau level. (a) The evolution of the wavefunctions in the Landau levels for a small force with δ = 0.01*g* (independent of the direction of the force). The total excitation number *N* = 20. In (b) and (c) we show the dynamics of the wavefunctions in the FSL with forces in the directions of the blue arrows. The red arrows show the directions of the velocities at τ = 0, *T*/2. The U-turn arrows show the velocity change before and after τ = *T*/4, 3*T*/4. The radii of the purple solid circles are proportional to the probabilities in the corresponding states.

(11) \begin{equation*} H_3=H_2+\delta \left(a_1^\dagger a_1-a_2^\dagger a_2\right), \end{equation*}

where δ is the detuning between the *a*_1_ and *a*_2_ modes. The direction of the effective force due to this potential is along the blue arrow in Fig. 4(b).

In Fig. 4, we prepare an initial state in the zeroth Landau level at the *K*′ valley, |ψ(0)〉 = |ψ_0, −*N*_〉, and show its dynamical evolution with Hamiltonian *H*_3_ by taking snapshots of the wavefunction at different times. The distributions of the states in both the energy bands and FSL are plotted. The electric field is small, δ ≪ *g*, such that Landau-Zener tunneling is negligible and the state stays in the zeroth Landau level. Driven by the effective electric field, the state moves from *K*′ to *K* (at time τ = *T*/2, where $T=\sqrt{3}\pi /\delta$) and then returns to point *K*′, as shown in Fig. 4(a), independent of the direction of the force. This is the Bloch oscillation in the zeroth Landau level. During this process, the most interesting feature of the valley Hall effect is demonstrated by the propagation of the wavefunction perpendicular to the direction of the force [28]. In Fig. 4(b) for a rightward force, the wavefunction moves upward at the *K*′ point (when τ = 0) and downward at the *K* point (when τ = *T*/2), which is unambiguous evidence that the pseudomagnetic fields at points *K* and *K*′ have opposite signs. This effect can also be demonstrated with forces in any other directions, e.g. upward as shown in Fig. 4(c) with the force term $\delta (a_1^\dagger a_1+a_2^\dagger a_2-2a_3^\dagger a_3)/\sqrt{3}$ in the Hamiltonian. Landau-Zener tunneling appears when the potential difference between neighboring lattice sites δ is comparable or larger than the band gap *g* (see the online supplementary material).

We can calculate the drift velocity in the limit of small electric field when δ ≪ *g* at the *K*′ point through the standard formula [45], e.g. for a horizontal force as shown in Fig. 4(b),

(12) \begin{equation*} v_D=\frac{\mathscr {E}}{B_0}=\frac{Nq\delta }{\sqrt{3}}, \end{equation*}

where $\mathscr {E}=2\hbar \delta /\sqrt{3}qe$ is the strength of the effective electric field. On the other hand, from an independent approach (see the online supplementary material), the drifted center of the wavepacket follows a sinusoidal oscillation with amplitude *R* = *Nq*/2 (the radius of the incircle of the triangular boundary),

(13) \begin{equation*} y(\tau )=R\sin {\frac{2\pi \tau }{T}}, \end{equation*}

where we have set the center of the lattice as the zero point and the coordinates *x* and *y* are defined in Equation (22) in the Methods section. We obtain the velocity

(14) \begin{equation*} v_y(\tau )\equiv \frac{dy(\tau )}{d \tau}=v_D\cos {\frac{2\pi \tau }{T}}. \end{equation*}

Obviously, at τ = 0 it coincides with the drift velocity obtained from Equation (12), $v$_*y*_(0) = $v$_*D*_. At τ = *T*/2, the wavepacket arrives at the *K* point and $v$_*y*_(*T*/2) = −$v$_*D*_.

Equations (13) and (14) also enable us to evaluate the strength of the pseudomagnetic field *B* away from the center of the lattice through $B(y)=\mathscr {E}/v_y(y)$. Because of the rotational symmetry of the Hall response in this lattice, from Equations (13) and (14) we obtain

(15) \begin{equation*} B^\pm (r)=\mp \frac{B_0}{\sqrt{1-r^2/R^2}\, }, \end{equation*}

where $r=\sqrt{x^2+y^2}$ is the distance to the center of the lattice, and *B*^+^(*r*) and *B*^−^(*r*) are for the *K* and *K*′ valleys, respectively. The distribution of *B*^−^(*r*) is plotted in Fig. 2(b) and the result is also consistent with a calculation based on the strain-induced shift of the Dirac cones (see the Methods section). In the *K*′ valley, the total number of magnetic flux quanta (Φ_0_ = 2πℏ/*e*) in the incircle of the FSL is ${\int _0^R 2\pi rB^-(r) dr}/{\Phi _0}={N}/{2}$, which means that *N*/2 states can be hosted in the *K*′ valley [21]. On the other hand, there are *N* + 1 eigenstates in the zeroth Landau level and half of them belong to the *K*′ valley, which is consistent with the above result from the total magnetic flux.

### The Haldane model in the 2D FSL

Although the 1D FSL is a topological SSH model, the 2D FSL has a topologically trivial Chern number, evident from the absence of gapless edge states. However, by introducing additional terms in the Hamiltonian, we can construct a Haldane model

(16) \begin{equation*} H_4=H_2+\kappa \sigma _z C/2, \end{equation*}

where κ is a coupling constant and the bosonic chirality operator *C* provides the next-nearest-neighbor coupling attached with a π/2 phase. The σ_$z$_*C* term can be synthesized by periodically modulating the frequencies of the cavities [22].

**Figure 5.**
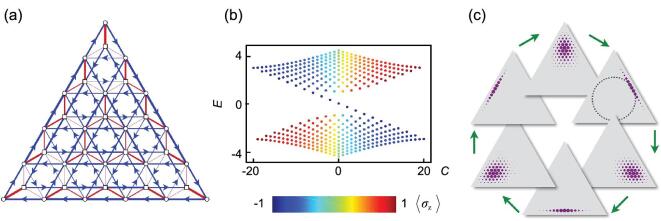
The Haldane model in the Fock-state lattice. (a) The coupling strengths of the Hamiltonian *H*_4_ in Equation (16) with *N* = 5. The nearest-neighbor couplings are denoted with red lines. The next-nearest-neighbor couplings are denoted by blue lines with arrows denoting the transition attached with a phase factor *i*. The linewidths are proportional to the coupling strengths. (b) The band structure of the Haldane model with *N* = 20, *g* = 1 and κ = 0.3 in *H*_4_. The color denotes the polarization of the eigenstates in |↑〉 (red) and |↓〉 (blue) components. (c) The dynamic evolution of a wavepacket of the edge states for *N* = 20, starting from the bottom frame. The dashed incircle denotes the trace of the weight (expectation value of the position) of this wavepacket during the evolution. The red arrows show the direction of time, sequentially at τ = *nT*_$w$_/6, where *T*_$w$_ = 2π/κ and *n* = 0, 1, 2, 3, 4, 5.

We plot the band structure of Equation (16) in Fig. 5(b). The bulk states in the conduction and valence bands are generated from the eigenstates $|\psi ^\pm _{m,C}\rangle$ in the Landau levels with *m* ≠ 0, and their eigenenergies are $E^{\pm }_{m,C}=\pm \sqrt{mg^2+\kappa ^2 C^2/4}$. The eigenstates in the zeroth Landau level turn into the chiral edge states with eigenenergies *E*_0, *C*_ = −κ*C*/2 connecting the *K* and *K*′ points of the two bands. The nontrivial topological property is demonstrated by the unidirectional propagation of a wave packet of the edge states [46,47], $\left| \psi (0)\right\rangle \,\,=\,\, (b_{+}^{\dagger }\,\,{-}\,\,b_{-}^{\dagger })^{N}{\left| \downarrow ,0,0,0\right\rangle _b}/{\sqrt{2^{N}N!}}\,\, =\break i^N(a_{1}^{\dagger }-a_{2}^{\dagger })^{N} {\left| \downarrow ,0,0,0\right\rangle }/{\sqrt{2^{N}N!}}$, which has zero mean energy. With the weight located on the incircle (the boundary between the band insulator and the semimetal), the wave packet rotates clockwise (as shown in Fig. 5(c)), which indicates the negative dispersion of the edge states in Fig. 5(b).

In the original Haldane model [2], the phase φ attached to the next-nearest-neighbor hopping can have values different from π/2 and there is an energy offset Δ between the two sublattices. A topological phase diagram can be plotted with respect to φ and Δ. The corresponding Hamiltonian in the FSL is

**Figure 6.**
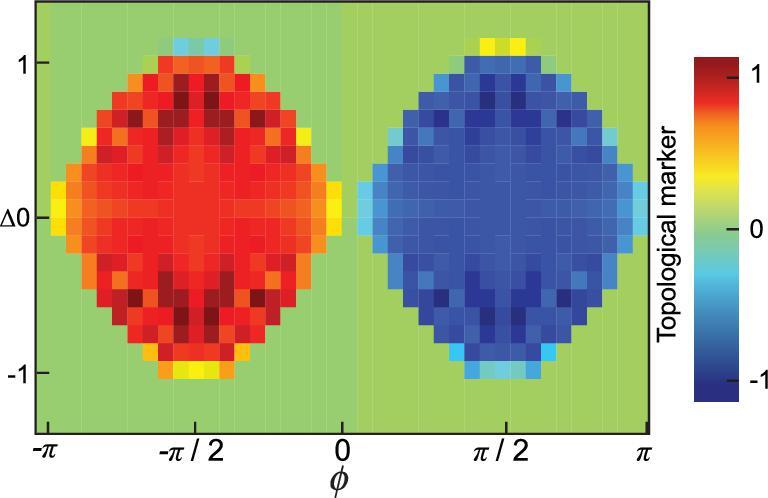
The topological marker of *H*_5_ in Equation (17) evaluated in the center of the Fock-state lattice. *g* = 0.05, $\kappa =2\sqrt{3}$ and *N* = 30.

(17) \begin{eqnarray*} \hspace*{-20pt}{H}_{5} &=& H_2+\frac{N\Delta }{2}\sigma _{z}\nonumber\\ &&+\left[\frac{\kappa }{2\sqrt{3}\, }e^{i\phi \sigma _z} \sum _{j=1}^{3}a_{j+1}^{\dagger }a_{j}{+}{\rm H.c.}\right]\!, \end{eqnarray*}

where Δ is the detuning between the frequencies of the cavities and that of the atom. The Chern numbers are traditionally obtained in the reciprocal space of lattices via a Bloch wavefunction in a closed Brillouin zone [48]. Since the FSL is finite with boundaries and nonuniform coupling strengths, the standard way to obtain the Chern number is not applicable. Instead, the Chern numbers of *H*_5_ are obtained through the local topological marker [31,32] in the center of the FSL (see the online supplementary material). They are plotted as a function of Δ and φ in Fig. 6, which demonstrates the same topological phase diagram as the original Haldane model [2].

### Topological quantum responses with coherent light field

The physics of topological quantum optics in the previous parts of the paper is based on the calculation with quantized Fock states. A natural question is whether some of these phenomena have classical correspondence and whether the topological properties can be observed with classical light. In particular, well-known classical phenomena of atom-light interactions shall be explained with the FSL. The quantum approach shall also give predictions that cannot be explained by classical optics. In the following we give an example to show the connection and difference between the semiclassical and quantum treatments.

In the semiclassical approach, the Hamiltonian of three classical light fields interacting with a single atom is (in the rotating frame with the rotating-wave approximation)

**Figure 7.**
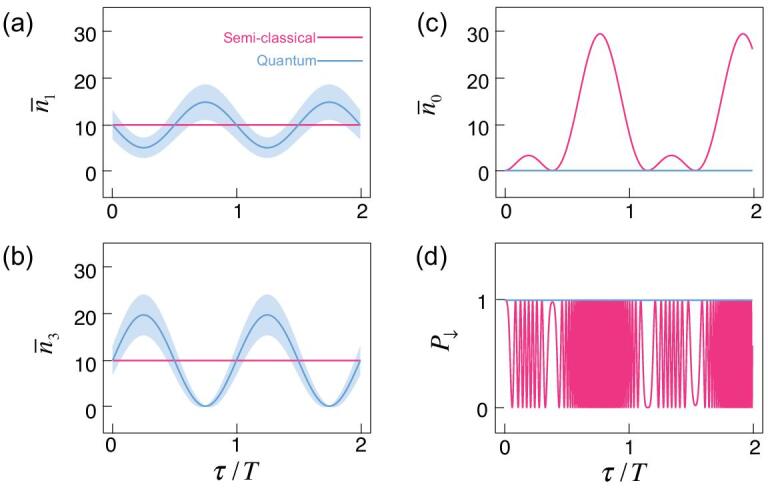
Evolution of the coherent cavity fields and atom with full quantum (blue lines) versus semiclassical (red lines) approaches. (a) The evolution of the average photon number $\bar{n}_1=\langle a_1^\dagger a_1\rangle$ (the same as that of mode *a*_2_). (b) The evolution of $\bar{n}_3=\langle a_3^\dagger a_3\rangle$. (c) The evolution of the photon number in the bright mode $\bar{n}_0=\langle b_0^\dagger b_0\rangle$, which is coupled with the atom. (d) The evolution of the ground-state population of the atom. The blue shaded areas in (a) and (b) show the uncertainties of the cavity photon numbers Δ*n*_1_ and Δ*n*_3_ in the quantum approach. Here *g* = 1, δ = 0.1 and |α|^2^ = 10. In addition to the quantum fluctuations, the quantum approach demonstrates nontrivial dynamic evolution of the cavity fields with the atom remaining in the ground state, while the semiclassical approach suggests Rabi oscillations of the atom and the photon numbers in the three cavities remain constant.

(18) \begin{equation*} H_c=\sum _{j=1}^3\Omega _je^{-i\Delta _j t-i\phi _j}\sigma ^+ +{\rm H.c.}, \end{equation*}

where the Ω_*j*_ are Rabi frequencies, and Δ_*j*_ = ν_*j*_ − ω and φ_*j*_ are the detunings and phases of the light modes. If Ω_*j*_ ≡ Ω, Δ_*j*_ = 0 for all modes and φ_*j*_ = 2*j*π/3, we obtain *H*_*c*_ = 0 since the three light fields cancel. The atom shall be decoupled with the cavities. The atom initially prepared in the ground state will remain there. We then introduce nonzero detunings Δ_1_ = δ and Δ_2_ = −δ, such that the atom interacts with a total light field Ω(*t*) = Ω[2cos (δ*t* + 2π/3) + 1]. The semiclassical treatment predicts that the atom shall be excited. The evolution of the total field and the atom is shown by the red lines in Fig. 7(c) and (d).

In the following we show that, when δ = 0, the semiclassical prediction of the decoupling between the atom and photons is consistent with the quantum prediction, i.e. it can be explained by the eigenstates in the zeroth Landau level of the FSL. However, when δ ≠ 0, in stark contrast to the semiclassical prediction, the quantum approach predicts that the atom stays in the ground state and the fields evolve in such a way that their amplitudes cancel out, as shown by the blue lines in Fig. 7(c) and (d). Then we make a transition to intrinsic topological quantum phenomena that can be demonstrated by a classical light field but without interpretation in classical optics.

The quantum state of the atom interacting with three classical light fields can be written as |↓, α_1_, α_2_, α_3_〉, where $| \alpha _j\rangle \,\,=\,\, \exp ({-|\alpha _j|^2/2)\sum _{n_j} \alpha _j^{n_j}| n_j\rangle }/\\\sqrt{n_j!}$ with the α_*j*_ being complex numbers are the coherent states of the cavity modes *a*_*j*_. The relative phases between the fields are taken into account by assuming that α_*j*_ = α exp (−*i*2*j*π/3) such that the three fields cancel, i.e. *b*_0_|↓, α_1_, α_2_, α_3_〉 = 0. This is consistent with the semiclassical prediction, i.e. the atom is decoupled from the fields since it experiences zero field strength. To understand this in the FSL, we find that the state can be expanded as a superposition of the eigenstates in the zeroth Landau levels of different subspaces (for |α|^2^ ≫ 1),

(19) \begin{eqnarray*} \!\!\!\!\!\!\!\left| \downarrow ,\alpha _1, \alpha _2, \alpha _3\right\rangle &=&\sum _N \frac{e^{{(N-N_0)}/{2}}}{(2\pi N)^{1/4}}\nonumber\\ &&\times\, \left(\frac{N_0}{N}\right)^{{N}/{2}} {\left|\psi _{0,-N}^{(N)}\right\rangle }, \end{eqnarray*}

where *N*_0_ = 3|α|^2^ is the total average photon number in the three modes and the $|\psi _{0,C}^{(N)}\rangle$ are the eigenstates in the zeroth Landau level of the subspace with total excitation number *N*. Here only the states at the *K*′ point appear and *C* = −*N*. Since these states are in the zeroth Landau levels, they are decoupled with the atom. In a more familiar form, the probability of obtaining $|\psi _{0,-N}^{(N)}\rangle$ in Equation (19) is approximately

(20) \begin{eqnarray*} &&\!\!\!\!\!\!\left|\langle \psi _{0,-N}^{(N)}\left| \downarrow ,\alpha _1, \alpha _2, \alpha _3\right\rangle \right|^2\nonumber\\ &&\approx\frac{1}{\sqrt{2\pi N}\, }e^{[-(N-N_0)^2/2N]}, \end{eqnarray*}

i.e. following a Gaussian distribution centered at *N*_0_. The result in Equation (19) is remarkable since it indicates that even with coherent fields in the three cavities we can prepare a state in the zeroth Landau level at the *K*′ point, although in a superposition of states from different subspaces. Since the Hamiltonian conserves *N*, the evolution of state |↓, α_1_, α_2_, α_3_〉 can be treated separately in each subspace.

Starting from the state in Equation (19), we show the difference between the predictions of semiclassical and full quantum approaches with the valley Hall effect in Fig. 7. In the semiclassical approach, a detuning δ (see Equation (11)) between the frequencies of modes *a*_1_ and *a*_2_ removes the canceling out of the three fields during the dynamical evolution, which predicts that the atom experiences a finite field strength and will be excited (see Fig. 7(d)). However, the excitation of the atom is absent in the quantum treatment for δ ≪ *g*. Instead, the state remains in the zeroth Landau level and the atom stays in the ground state (see the online supplementary material). This intraband evolution is protected by the band gap *g*, which is also the vacuum Rabi splitting and the cyclotron frequency in the pseudomagnetic field.

On the other hand, the cavity modes undergo a nontrivial evolution. Without the atom, the three fields do not interact with each other. With the presence of the atom, the valley Hall effect induces exchange of photons between the three cavity modes such that the zero value of their superposition is maintained, as shown in Fig. 7(a)–(c). The bright mode *b*_0_ is a dynamical constant (i.e. it commutes with the effective Hamiltonian equation (S7) in the online supplementary material). Note also that in order to keep *b*_0_ zero, a classical version of the relation in Equation (5), ||α_1_| − |α_2_|| ≤ |α_3_| ≤ |α_1_| + |α_2_|, must be satisfied, which is also consistent with the fact that the wavepacket is trapped within the incircle of the FSL (see Fig. 4). For instance, at time τ = 3*T*/4, the state in Equation (19) evolves to $|\downarrow,-i\sqrt{6}\alpha/2,i\sqrt{6}\alpha/2,0\rangle$, i.e. the cavity mode *a*_3_ is in the vacuum state and the photons are equally distributed in modes *a*_1_ and *a*_2_. Therefore, the topological quantum phenomena discussed in this paper can be observed with the classical (coherent) field, but they cannot be explained with classical optics. Similarly, the dynamics of the edge states of the Haldane model in Fig. 5(c) can also be demonstrated with coherent light fields.

## CONCLUSION

In striking contrast to the photonic and acoustic topological insulators [8,9,12,49], where the topological properties do not require a quantization of the light field, all the topological properties discussed in this paper are based on the quantum nature of the bosonic operator, i.e. $a\left| n\right\rangle =\sqrt{n}\left| n-1\right\rangle$ for *n* ≥ 1 and *a*|0〉 = 0 (which ensures finite lattices with edges). Another difference from the photonic and acoustic topological insulators is that the FSL needs only a few modes to generate high-dimensional lattices. We can use *d* bosonic modes to construct an FSL in *d* − 1 dimensions, which offers a platform to simulate high-dimensional topological physics [33–37].

This study can also help to design novel artificial lattices for photons and phonons. A special type of lattice named the Glauber-Fock lattice [50,51] has been fabricated with waveguides, with the coupling strengths between neighboring waveguides mimicking the coupling between Fock states. These lattices can host collective modes that inherit the properties of the coherent state. In the same spirit, by replacing each state in the FSL with a cavity mode, we can construct a finite lattice of cavities that has a band structure similar to that in Fig. 3(c), with each eigenstate being replaced by an eigenmode. Compared with the lattices designed with the strain-induced gauge field [38,39,43,44], the lattice with coupling strengths between neighboring sites mimicking the 2D FSL has $\sqrt{m}$-scaling quantized energy levels everywhere, not limited near the *K* and *K*′ points.

The experimental realization of the physics discussed in the paper can be implemented in superconducting circuits with several resonators being coupled to a single qubit. In order to observe the dynamical process of the valley Hall effect and the chiral edge states of the Haldane model, we need the lifetime of the resonator *T*_*R*_ to satisfy *T*_*R*_/*N* ≥ *T*, *T*_$w$_. Since only the zeroth Landau level with the qubit in the ground state is involved with these two phenomena, the decoherence from the qubit has no effect. For Landau-Zener tunneling, the atom can be in the excited state and thus it also requires *T*_*a*1_, *T*_*a*2_ ≥ *T*, *T*_$w$_, where *T*_*a*1_ and *T*_*a*2_ are the lifetime and decoherence time of the qubit. The state-of-the-art parameters are *T*_*R*_ ≈ 20 μs, *T*_*a*1_ ≈ 20 μs, *T*_*a*2_ ≈ 2 μs, *g* ≈ 2π × 50 MHz [52] and *T*_$w$_ ≈ 450 ns [53]. If we adopt a reasonable *T* = 200 ns for δ = 2π × 5 MHz, the above conditions can be satisfied with excitation number *N* = 10, which is sufficient to observe the topological phenomena.

## METHODS

### The Lifshitz topological phase transition in the FSL

It has been shown that the strain can shift the Dirac cones of graphene, which has the effect of a vector potential until the anisotropy of the strain is large enough to merge two Dirac cones into one, beyond which a band gap opens [27]. Here we show that the Lifshitz topological phase transition happens at the incircle of the triangular boundary of the FSL. Considering the lattice site |↓, *n*_1_, *n*_2_, *n*_3_〉, the coupling strengths are $t_j=\sqrt{n_j}g/\sqrt{3}$. The condition for the semimetallic phase in Equation (5) can be rewritten as

(21) \begin{equation*} n_1+n_2-2\sqrt{n_1n_2}<n_3<n_1+n_2+2\sqrt{n_1n_2}. \end{equation*}

The *x* and *y* coordinates in the Fock-state lattice are

(22) \begin{eqnarray*} y& =& \frac{q}{2}\left(2a_{3}^{\dagger }a_{3}-a_{1}^{\dagger }a_{1}-a_{2}^{\dagger }a_{2}\right),\nonumber\\ x& =& \frac{\sqrt{3} q}{2} \left(a_{2}^{\dagger }a_{2}-a_{1}^{\dagger }a_{1}\right). \end{eqnarray*}

From Equation (22) and the constraint ∑ _*j*_*n*_*j*_ = *N*, we obtain

(23a) \begin{equation*} n_1=\frac{Nq-y}{3q}-\frac{x}{\sqrt{3}q}, \end{equation*}

(23b) \begin{equation*} n_2=\frac{Nq-y}{3q}+\frac{x}{\sqrt{3}q}, \end{equation*}

(23c) \begin{equation*} n_3=\frac{N}{3}+\frac{2y}{3q}.\hspace*{2.9pc} \end{equation*}

Substituting Equation (23) into Equation (21), we obtain

(24) \begin{equation*} x^2+y^2<R^2, \end{equation*}

i.e. the sites are in the incircle of the triangular boundary. Substituting Equation (22) into Equation (24), we obtain the relation of the photon numbers in Equation (6).

### Pseudomagnetic field obtained from the shift of Dirac points

At the Dirac points of a tight-binding honeycomb lattice, the Bloch wavevectors $\mathbf {k}$ satisfy the relation

(25) \begin{equation*} |t_{3}+t_{1}e^{-i\mathbf {k}\cdot \mathbf {v}_{1}}+t_{2}e^{-i\mathbf {k}\cdot \mathbf {v}_{2}}|=0, \end{equation*}

where $\mathbf {v}_{1}=(-\sqrt{3}q/2,-3q/2)$ and $\mathbf {v}_{2}=(\sqrt{3}q/2,-3q/2)$. Accordingly, the positions of the Dirac points are explicitly obtained through the equations

(26) \begin{eqnarray*} \cos \mathbf {k}\cdot \mathbf {v}_{1}&=&\frac{t_{2}^{2}-t_{1}^{2}-t_{3}^{2}}{2t_{1}t_{3}}\equiv s_{1},\nonumber\\ \cos \mathbf {k}\cdot \mathbf {v}_{2}&=&\frac{t_{1}^{2}-t_{2}^{2}-t_{3}^{2}}{2t_{2}t_{3}}\equiv s_{2}. \end{eqnarray*}

In the FSL the coupling strengths vary locally and the Dirac points shift at different locations. At the site |↓, *n*_1_, *n*_2_, *n*_3_〉, we obtain

(27) \begin{eqnarray*} s_1&=&\frac{n_{2}-n_{1}-n_{3}}{2\sqrt{n_{1}n_{3}}},\nonumber\\ s_2&=&\frac{n_{1}-n_{2}-n_{3}}{2\sqrt{n_{2}n_{3}}}. \end{eqnarray*}

From Equation (26) we obtain

(28) \begin{eqnarray*} k^\pm _{x}&=&\pm \frac{1}{\sqrt{3}q}(\arccos s_{1}+\arccos s_{2}),\nonumber\\ k^\pm _{y}&=&\pm \frac{1}{3q}(\arccos s_{1}-\arccos s_{2}), \end{eqnarray*}

where $\mathbf {k}^\pm =(k^\pm _x, k^\pm _y)$ with + and − denoting the two Dirac points *K* and *K*′. The Hamiltonian near the Dirac points can be written as $H^\pm =v_F(\mathbf {p}-\hbar \mathbf {k}^\pm )\cdot \mathbf {\sigma }$, where $v_F=gq\sqrt{N}/2$ is analogous to the Fermi velocity in graphene [21] and $\mathbf {p}$ is the canonical momentum. Comparing *H*^±^ with the minimal coupling Hamiltonian $v_F(\mathbf {p}-e\mathbf {A}^\pm )\cdot \mathbf {\sigma }$, we obtain the pseudovector potential

(29) \begin{equation*} \mathbf {A}^\pm =\left(A_{x}^{\pm },A_{y}^{\pm }\right)=\frac{\hbar }{e}(k^\pm _{x},k^\pm _{y}), \end{equation*}

which results in the following pseudomagnetic field for *N* ≫ 1:

(30) \begin{eqnarray*} B^{\pm }&=&\frac{\partial A_{y}^{\pm }}{\partial x}-\frac{\partial A_{x}^{\pm }}{\partial y}\nonumber\\ &=&\frac{\hbar }{e}\left(\frac{\partial k_{y}^{\pm }}{\partial x}-\frac{\partial k_{x}^{\pm }}{\partial y}\right). \end{eqnarray*}

Substituting Equations (23), (27) and (28) into Equation (30) and after a cumbersome algebraic calculation, we obtain

(31) \begin{eqnarray*} B^{\pm } & =& \mp \frac{2\hbar }{Neq^{2}}\frac{1}{\sqrt{1-4r^{2}/q^2N^{2}}\, } \nonumber \\ & =&\mp \frac{B_{0}}{\sqrt{1-r^{2}/R^{2}}\, }, \end{eqnarray*}

which is consistent with that obtained from the valley Hall effect in Equation (15).

## Supplementary Material

nwaa196_Supplemental_File
