# Supplementary material for: Topological phases of quantized light
Source: Natl Sci Rev. 2020 Aug 31;8(1):nwaa196. doi: 10.1093/nsr/nwaa196 (PMC8288454; doi:10.1093/nsr/nwaa196)
Supplement: nwaa196_Supplemental_File [file nwaa196_supplemental_file.pdf]

National Science Review
1: 1–?, 2020
doi: ??????????
Advance access publication 24 December 2013

RESEARCH ARTICLE

Topological phases of quantized light: Supplementary data

Han Cai[1] & Da-Wei Wang[1,2*]

[1]Interdisciplinary Center for Quantum Information and State Key Laboratory of Modern Optical Instrumentation, Zhejiang Province Key Laboratory of Quantum Technology and Device and Department of Physics, Zhejiang University, Hangzhou 310027, China;
[2]CAS Center for Excellence in Topological Quantum Computation, University of Chinese Academy of Sciences, Beijing 100190, China;
Corresponding authors E-mail: dwwang@zju.edu.cn

ABSTRACT
This document provides supplementary information to "Topological phases of quantized light", National Science Review volume, first page (year). The location of the zero-energy state in the 1D Fock-state SSH model and the wavefunctions in the zeroth Landau level of 2D FSL are calculated. We define the bosonic chirality operator to characterize the degenerate eigenstates in the same Landau levels. The valley Hall response and the Landau-Zener tunneling are calculated and numerically simulated. We also introduce the topological marker to identify the topological phases of the Haldane model in the FSL.

Keywords topological phases, Su-Schriefer-Heeger model, Jaynes-Cummings model, strain-induced gauge field, valley Hall effect, Haldane model

Location of the topological zero-energy state in 1D Fock-state SSH model

We expand $|\psi_s\rangle$ in the basis of a modes,

$$|\psi_s\rangle = \sqrt{\frac{N!}{n_1!n_2!}} u_2^{n_1}(-u_1)^{n_2}|\downarrow, n_1, n_2\rangle, \tag{S1}$$

according to which Fig. 2 (a)-(c) are drawn. The probability distribution of the dark state in Eq. (S1) is

$$|\langle\downarrow, n_1, n_2|\psi_s\rangle|^2 \propto \frac{u_2^{2n_1}u_1^{2n_2}}{n_1!n_2!}. \tag{S2}$$

By using the Stirling's formula for $N \gg 1$ and $1 \ll n_1 \ll N$, we obtain the condition of the distribution maxima in the lattice,

$$\frac{\partial}{\partial n}\ln|\langle\downarrow, n_1, n_2|\psi_s\rangle|^2 \propto \ln\left(\frac{u_2^2}{u_1^2}\frac{n_2}{n_1}\right) = 0, \tag{S3}$$

which results in

$$u_1\sqrt{n_1} = u_2\sqrt{n_2}, \tag{S4}$$

i.e., the state is centered at the point where the two neighboring coupling strengths are equal, and the photon number in a_1 mode is $n_1 = u_2^2 N$.

The eigen wavefunction in the zeroth Landau level

Here we compare the wavefunction in the zeroth Landau level near K' point $|\psi_{0,-N}\rangle$ (see Fig. 3 (d)) with that in the Landau level of a real magnetic field in the symmetric gauge, $\psi_{0LL}(r) \propto \exp(-r^2/4l_B^2)$ [1]. We will show that $|\psi_{0,-N}\rangle$ is more localized in the Fock-state lattice than $\psi_{0LL}(r)$ due to the inhomogeneity of the magnetic field (see Fig. 3 (b) and Eq. (15)). The probability distribution of $|\psi_{0,-N}\rangle$ is

$$|\langle\downarrow, n_1, n_2, n_3|\psi_{0,-N}\rangle|^2 = \frac{N!}{3^N n_1!n_2!n_3!}, \tag{S5}$$

which has rotational symmetry. Therefore, we only need to consider the distribution along a radial direction from the center of the lattice to vertex 1 in Fig. 3 (a), e.g., in states $|p\rangle \equiv |\downarrow, N/3-2p, N/3+p, N/3+p\rangle$ where $-N/3 \leqslant p \leqslant N/6$ being an integer. By using the Stirling's formula for $N \gg 1$ and $p \ll N$, we obtain

$$|\langle p|\psi_{0,-N}\rangle|^2 \propto \exp\left(-\frac{9p^2}{N}\right) = \exp\left(-\frac{2r^2}{l_B^2}\right), \tag{S6}$$

where the radius r is related to number p through $r = 3pq/2$. Comparing with $|\psi_{0LL}(r)|^2 \propto \exp(-r^2/2l_B^2)$, $|\psi_{0,-N}\rangle$ has a smaller variance.

It has been shown that in graphene the wavefunctions at K and K' points have phase winding of $0, 2\pi/3, -2\pi/3$ for three neighboring lattice sites in the same sublattices [2]. We show that this phase winding also exists in $|\psi_{0,\pm N}\rangle$ (see Fig. S1).

Bosonic chirality operator

The spin chirality operator is defined for three spins, $C_s = \vec{\sigma}_1 \cdot (\vec{\sigma}_2 \times \vec{\sigma}_3)$ where $\vec{\sigma}_j$ is the Pauli matrix vec-

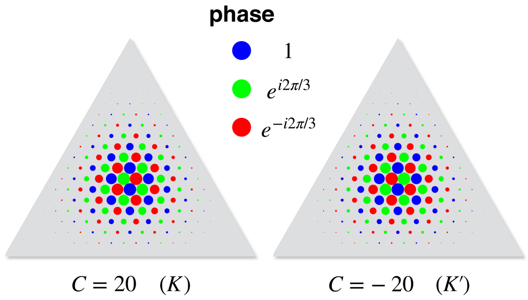

Figure S1 The phase distribution of the wavefunctions at the K and K' points for a FSL with $N = 20$. The phase winding of the state $|\psi_{0,N}\rangle$ on the K point is opposite to the one of $|\psi_{0,-N}\rangle$ at the K' point.

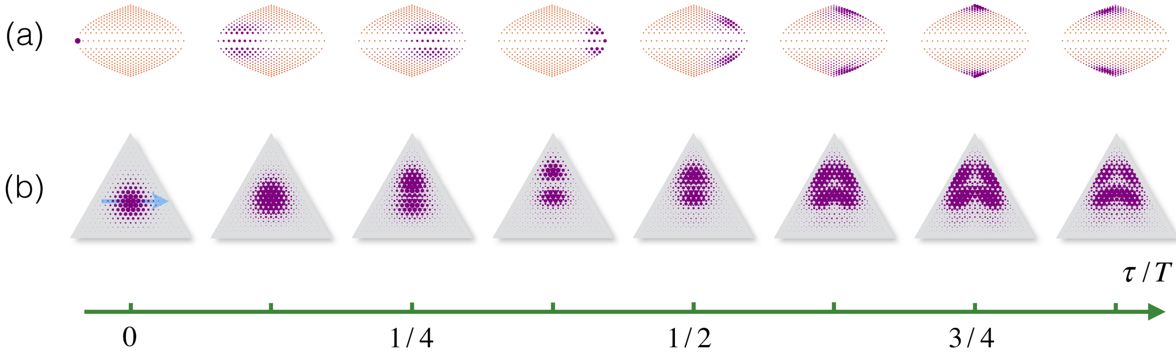

Figure S2 The Landau-Zener tunneling in the Fock-state lattice with $N = 20$. (a) The Landau-Zener tunneling shown by the wavefunctions spreading to higher Landau levels due to a large effective force, $\delta = g$, in the direction shown by the blue arrow. (b) The evolution of the wavefunctions in the FSL. The radii of the purple solid circles are proportional to the probabilities in the corresponding states.

tor of the jth spin. To generalize the chirality operator to bosons, we need to notice that the spin chirality operator breaks both the parity $\mathscr{P}$ and time-reversal $\mathscr{T}$ symmetry, but conserves the $\mathscr{P}\mathscr{T}$ symmetry [3]. Another key feature of the spin chirality operator is that its evolution operator chirally rotates the spin states, $|s_1 s_2 s_3\rangle \rightarrow |s_2 s_3 s_1\rangle \rightarrow |s_3 s_1 s_2\rangle$ [4]. The chirality operator defined in Eq. (9) has these two properties. It is easy to verify that $\mathscr{P}C\mathscr{P}^{-1} = -C$, $\mathscr{T}C\mathscr{T}^{-1} = -C$ and $\mathscr{P}\mathscr{T}C\mathscr{T}^{-1}\mathscr{P}^{-1} = C$. The evolution operator of C rotates photons chirally among the three modes $a_1 \rightarrow a_2 \rightarrow a_3$ [5].

The valley Hall response

In the limit of a small effective electric field, $\delta \ll g$, the evolution of an initial state in the zeroth Landau level is confined in that level. We can study the evolution by projecting the Hamiltonian H_3 in Eq. (11) to the subspace of the zeroth Landau level,

$$\begin{aligned} H_{\text{eff}} &= P_0 H_3 P_0, \\ &= \frac{\delta}{\sqrt{3}}(i b_+^\dagger b_- - i b_-^\dagger b_+), \end{aligned} \tag{S7}$$

where $P_0 = \sum_C |\psi_{0,C}\rangle\langle\psi_{0,C}|$ is the projection operator in the zeroth Landau level. The Heisenberg equations of the operators are

$$\begin{aligned} \frac{d}{d\tau} b_+^\dagger &= i[H_{\text{eff}}, b_+^\dagger] = \frac{\delta}{\sqrt{3}} b_-^\dagger, \\ \frac{d}{d\tau} b_-^\dagger &= i[H_{\text{eff}}, b_-^\dagger] = -\frac{\delta}{\sqrt{3}} b_+^\dagger. \end{aligned} \tag{S8}$$

We obtain the state evolution

$$\begin{aligned} b_+^\dagger(0) &= \cos(\frac{\delta}{\sqrt{3}}\tau) b_+^\dagger(\tau) - \sin(\frac{\delta}{\sqrt{3}}\tau) b_-^\dagger(\tau), \\ b_-^\dagger(0) &= \sin(\frac{\delta}{\sqrt{3}}\tau) b_+^\dagger(\tau) + \cos(\frac{\delta}{\sqrt{3}}\tau) b_-^\dagger(\tau). \end{aligned} \tag{S9}$$

The evolution of the state $|\downarrow, 0, 0, N\rangle_b$ at K' point is determined by

$$\frac{[b_-^\dagger(0)]^N}{\sqrt{N!}} |\downarrow, 0, 0, 0\rangle_b = \sum_n c_{n,N-n}(\tau) |\downarrow, 0, n, N-n\rangle_b, \tag{S10}$$

where $|c_{n,N-n}(\tau)| \propto |(\cos\frac{\delta}{\sqrt{3}}\tau)^n (\sin\frac{\delta}{\sqrt{3}}\tau)^{N-n}|$ can be obtained through Eq. (S9) and the distribution is shown in Fig. 4 (a), demonstrating the Bloch oscillation in the zeroth Landau level,

$$|\downarrow, 0, 0, N\rangle_b \xrightarrow{T/2} |\downarrow, 0, N, 0\rangle_b \xrightarrow{T/2} |\downarrow, 0, 0, N\rangle_b. \tag{S11}$$

The x and y coordinates in the Fock-state lattice are

$$
\begin{aligned}
y &= \frac{q}{2}(2a_3^\dagger a_3 - a_1^\dagger a_1 - a_2^\dagger a_2), \\
x &= \frac{\sqrt{3}q}{2}(a_2^\dagger a_2 - a_1^\dagger a_1).
\end{aligned} \tag{S12}
$$

Since x commutes with H_{eff}, it does not change with time. This is a signature of the Hall response considering that the force is along the x direction. Using Eq. (S12) and the inverse relation of Eq. (S9) and considering the initial state $|\downarrow, 0, 0, N\rangle_b$, we obtain the evolution in the y direction,

$$
\begin{aligned}
y(\tau) =& \frac{q}{2}\langle b_-^\dagger(\tau)b_+(\tau) + b_+^\dagger(\tau)b_-(\tau)\rangle \\
=& \frac{q}{2}\langle b_-^\dagger(0)b_-(0) - b_+^\dagger(0)b_+(0)\rangle \sin\frac{2\delta\tau}{\sqrt{3}} \\
=& R\sin\frac{2\pi\tau}{T}.
\end{aligned} \tag{S13}
$$

The Landau-Zener tunneling and the wavefunctions beyond the zeroth Landau level

The Landau-Zener tunneling appears when the potential difference between neighboring lattice sites δ is comparable with the bandgap g, as shown in Fig. S2. While oscillating between the K and K' points, the state tunnels to other Landau levels. In the FSL, the Landau-Zener tunneling add new features such as the splitting of the wavepackets into multiple components, demonstrating interference between states in different Landau levels.

The eigenstates in the zeroth Landau level of the 2D FSL is a two-dimensional extension of the topological zero-energy states in the 1D FSL. In particular, the weight of a wavepacket centered at the state $|\psi_{0,0}\rangle$ locates on the incircle, as shown by Fig. 5. Therefore, we can use $|\psi_{0,0}\rangle$ to set the boundary between the eigenstates in the strained semimetal and the band insulator. In Fig. S3, the eigenstates with variations $\langle r^2\rangle$ smaller than that of $|\psi_{0,0}\rangle$ are plotted red, otherwise blue, which can be regarded as in two different topological phases. Several typical eigen wavefunctions are also plotted. The one in the blue area occupies more sites outside of the incircle.

Topological marker

We calculate the local topological marker in the filled hexagon in Fig. S4 according to ref. [6],

$$
M = \frac{8\pi}{9\sqrt{3}q^2}\mathrm{Im}\sum_{s\in\hexagon}\langle s|\, P_- x P_- y P_-\, |s\rangle \tag{S14}
$$

where $|s\rangle$ are the Fock states in the unit cell colored in Fig. S4, $P_- = \sum_{m,C}|\psi_{m,C}^-\rangle\langle\psi_{m,C}^-|$ is the projection operator of the lower band where the eigenstate

$$
\begin{aligned}
|\psi_{m,C}^-\rangle =& -\sin\frac{\theta}{2}\,|\downarrow, m, m_+, m_-\rangle_b \\
&+ \cos\frac{\theta}{2}\,|\uparrow, m-1, m_+, m_-\rangle_b\,,
\end{aligned} \tag{S15}
$$

with $\tan\theta = \kappa C/2g\sqrt{m}$, x and y are position operators in Eq. (S12). The topological marker M is plotted in Fig. 6 for the phase diagram of the FSL Haldane model.

REFERENCES

1. Z. F. Ezawa, *Quantum Hall Effects: Field Theoretical Approach and Related Topics, Second Edition.* World Scientific Publishing Company, 2008.
2. R. Rammal, *Landau level spectrum of Bloch electrons in a honeycomb lattice.* J. Physique 46, 1345-1354 (1985).
3. X. G. Wen, F. Wilczek, and A. Zee, *Chiral spin states and superconductivity.* Phys. Rev. B **39**, 11413-11423 (1989).
4. D. W. Wang, C. Song, W. Feng, H. Cai, D. Xu, H. Deng, H. Li, D. Zheng, X. Zhu, H. Wang, S. Y. Zhu, and M. O. Scully, *Synthesis of antisymmetric spin exchange interaction and chiral spin clusters in superconducting circuits.* Nat. Phys. **15**, 382-386 (2019).
5. D. W. Wang, H. Cai, R. B. Liu, and M. O. Scully, *Mesoscopic superposition states generated by synthetic spin-orbit interaction in Fock-state lattices.* Phys. Rev. Lett. **116**, 220502 (2016).
6. R. Bianco and R. Resta, *Mapping topological order in coordinate space.* Phys. Rev. B **84**, 241106 (2011).

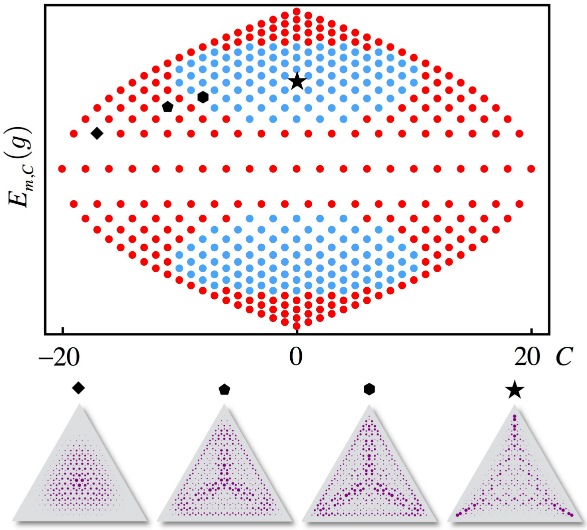

Figure S3 The eigenstates in (red) and out of (blue) the incircle. Four typical wavefunctions in the first (diamond), third (pentagon), fourth (hexagon) and sixth (star) Landau levels are plotted in the FSL.

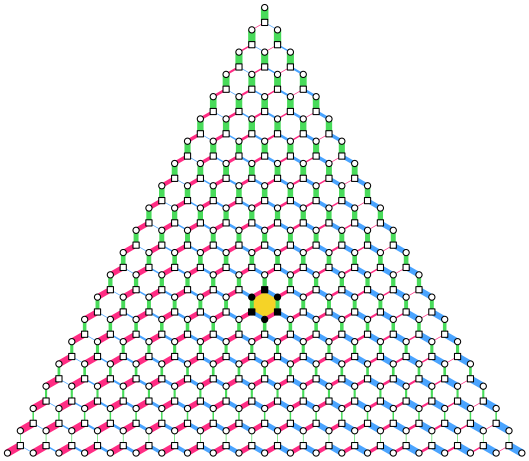

Figure S4 The Fock-state lattice (with $N = 20$) where the topological marker is calculated in the colored unit cell with the six vertices filled with black color.
